# We Must Not Let Protestors Derail Trials of Pre-Exposure Prophylaxis for HIV

**DOI:** 10.1371/journal.pmed.0020248

**Published:** 2005-07-19

**Authors:** Joep M. A Lange

## Abstract

The international AIDS community is being held hostage by a small number of activist groups, argues Lange.

One of the great tragedies of our times is the extent to which HIV prevention efforts are falling short. In 2004, more new HIV infections occurred than in any previous year: close to 14,000 a day, 570 per hour, almost ten per minute. The greater part of new infections occurs in young people, over half in persons between 15 and 24 years of age, and over half in women. The increasing feminization of the HIV/AIDS epidemic reflects the vulnerable position of women in many societies [1]. HIV is a virus, but inequity is at the roots of most of its spread.

Condoms are highly effective at preventing sexual transmission of HIV, but only if they are available and used [2]. Even if the former is the case, women are often in a difficult position to negotiate use by their male partners [3]; this applies to female as well as male condoms.

In the absence of an effective preventive HIV vaccine [4], which is felt to be the only tool that can definitively break the epidemic, there is thus great need for alternative prevention technologies, especially those that can be “female-controlled”, i.e., use of which does not require consent of the male partner. Other vulnerable populations might equally profit from the availability of such interventions. The urgent need to develop female-controlled prevention methods explains the thrust to develop vaginal microbicides [5,6], and the more recent interest in using an antiretroviral oral pre-exposure prophylaxis (PREP), for which proof of concept has come from preventing mother-to-child transmission via breastfeeding [7].

Mills and Singh have written an informative and thoughtful essay for *PLoS Medicine* on the recent interruptions of several tenofovir PREP trials, instigated predominantly by activist groups, including Act Up–Paris [8]. They properly describe what has happened in the countries concerned, and, at least initially in their essay, they do not seem to be afraid of identifying culprits, albeit in an indirect way. For example, they say, “while freedom of expression is a cherished ideal, protest should be carried out in a responsible manner”, and “speculation, unwarranted criticism, overreaction, or sensationalizing facts risks stigmatizing tenofovir”. But I feel that in the end, the authors could have gone further in their criticism of the protestors who derailed the tenofovir trials and in their support for the trial investigators and sponsors.

The fear or trepidation about making a correct diagnosis, or being outspoken about the correct diagnosis, is in this case, as in most other circumstances, counterproductive and may even lead to more harm being done. The fact of the matter is that the investigators of the tenofovir trials did consult intensely with community groups concerned, but this consultative process obviously did not include every activist group in the world.

As Co-Chair of last year's XVth International AIDS Conference in Bangkok, Thailand, I became painfully aware of a structural flaw in the system of dealing with the activist community. The governing body of the conference, the Conference Organizing Committee, included three international and one local Community Co-Organizers, who were supposed to have a mandate to speak on behalf of the national and international AIDS community. In the end, though, it turned out that agreements on limiting disruption of speeches and destruction of property, which took endless hours of discussion and which were communicated to attendants, governments, speakers, and sponsors, could not be honored because certain activist groups simply ignored them. Likewise, in clinical research, investigators may end up in situations in which they may have had an intense dialogue and come to an agreement with what they have identified to be the relevant community organizations, and yet a day later are put on the stand by yet another activist group.

Life would be simpler if there were an umbrella organization with undisputed leadership among AIDS activists that was mandated to speak and act on behalf of this community; somewhat like labor unions in their heydays. But we are far from that.

Activist groups have now managed to derail several PREP trials, arguably the most important studies for those at high risk of acquiring HIV infection around the globe. Similarly, activist groups have endangered the funding and therefore the continuity of the International AIDS Conference (the only global forum about HIV/AIDS where researchers, health-care workers, community, activists, drug manufacturers, politicians, and leaders from all walks of life meet). And lately activist groups have prevented clinical trials with the promising and highly needed new class of CCR5-receptor-blocking antiretrovirals from proceeding in several European countries.

The methods of these specific activist groups are uninformed demagogy, intimidation, and “AIDS exceptionalism”, the last in the sense that they exploit their HIV-positive status to get away with behavior that would not be accepted from others. Within the international AIDS community, such form of activism is only practiced by a tiny minority, but it has taken us hostage. Those who will suffer the most from the misguided ethical imperialism that derailed the PREP trials do not live in Paris, but as usual in Nairobi, Johannesburg, Phnom Penh, and Calcutta.

There is no other area of medicine where activism has been so strong and has accomplished so much as in the HIV/AIDS field. Let's be just a little brave, and stand up to protect that legacy.

## Abbreviations

PREP: pre-exposure prophylaxis
